# Examining psychosocial risks and their impact on nurses' safety attitudes and medication error rates: A cross-sectional study

**DOI:** 10.3934/publichealth.2025022

**Published:** 2025-03-20

**Authors:** Vasileios Tzenetidis, Aristomenis Kotsakis, Mary Gouva, Konstantinos Tsaras, Maria Malliarou

**Affiliations:** 1 Laboratory of Education and Research of Trauma Care and Patient Safety; Department of Nursing, University of Thessaly, Larissa, Greece; 2 Department of Management Science and Technology, University of Patras, Patras, Greece; 3 Nursing Department, University of Ioannina, Ioannina, Greece; 4 Department of Nursing, University of Thessaly, Larissa, Greece

**Keywords:** nurses, psychosocial risks, medication errors, patient safety

## Abstract

**Introduction:**

Employee exposure to specific risks often increases work-related stress, negatively impacting their effectiveness and potentially leading to illnesses, mistakes, or accidents.

**Objective:**

We aimed to determine the psychosocial risks experienced by nurses in tertiary hospitals and their association with attitudes toward safety and the occurrence of medication errors.

**Methods:**

A cross-sectional study was conducted between September 30, 2022 and December 31, 2023 in four Greek tertiary hospitals (Evangelismos, Nikaia “Agios Panteleimon”, University Hospital of Larissa, and “G. Papanikolaou”). The study involved 514 nurses aged 20–67, employed for at least 12 months, fluent in Greek, and completing questionnaires on stress, burnout, and medication errors. The questionnaire used in the study included demographic information of the nursing staff (age, gender, marital status, work experience, and education level), characteristics of the nursing unit (medical, surgical, long-term care unit, Intensive Care Unit), the COPSOQ III (Copenhagen Psychosocial Questionnaire Version III), the HSOPSC (Hospital Survey on Patient Safety Culture), and the questionnaire for Investigating Nursing Errors in Medication Administration.

**Results:**

Nurses exposed to psychosocial risks, such as bullying and high demands, reported increased medication errors. Supportive work environments with sufficient staffing and collaborative culture significantly mitigated these risks. Factors such as “Staffing” and “Handoffs” partially mediated the relationship between demands and errors. Thus, targeted interventions to reduce bullying and enhance teamwork are essential. Continuous education emerged as crucial for improving safety and performance.

**Conclusion:**

The study underscores the necessity of social support, job autonomy, and work-life balance as critical factors in reducing stress and improving the quality of care. Specific strategies are proposed to enhance nurses' mental health and improve working conditions.

## 1. Introduction

In modern workplaces, maintaining a balance between job demands and employee well-being has become a key research focus. As organizations pursue greater efficiency and productivity, employees often face increased workloads and pressures. These conditions can lead to psychosocial risks that affect not only individual health but also organizational performance. Addressing these risks is vital for fostering a healthier and more sustainable work environment [1]. Workplaces can impact health in several ways, with workplace strain being the second most common health issue in the European Union, affecting approximately one-third of employees [2],[3]. Workplace strain encompasses factors such as physical fatigue, mental pressure, work-related stress, and emotional exhaustion. These stressors can significantly harm employees' physical and mental health. At an organizational level, the negative impacts include poor business performance, increased absenteeism, presenteeism (where employees work despite being unwell), and higher accident and injury rates [4]. The World Health Organization defines health as a state of complete physical, mental, and social well-being, extending beyond the mere absence of disease or disability. This definition integrates biological needs, mental balance, and social participation [5]. Under this broader understanding, health involves not just medical care but also psychosocial support and social justice, promoting overall community well-being [6]. The nursing profession is particularly demanding, with high levels of both mental and physical strain, often resulting in occupational diseases and injuries. The work environment plays a crucial role in the health and safety of nurses, making it one of the most hazardous professions globally [7],[8]. Contributing factors to this strain include physical labor, long hours without adequate breaks, and the stress of managing emergency situations. These conditions increase the risk of both physical injuries, such as musculoskeletal problems, and mental health issues like anxiety, depression, and professional burnout [9]. Among the most significant risks for nurses are psychosocial hazards such as work strain, bullying, burnout, emotional strain, and work-life imbalance [10]–[14]. These factors contribute to job dissatisfaction, intention to leave, and various mental health issues [12],[15],[16]. Nurses are also frequently victims of verbal and physical violence from patients or their relatives, which creates a sense of insecurity and can lead to long-term mental health problems [17],[18]. Contributing factors to this strain include physical labor, long hours without adequate breaks, and the stress of managing emergency situations. Achieving a healthy work-life balance is challenging for nurses, as long shifts, frequent overtime, and limited personal time create additional stress, further exacerbating workplace strain [19],[20].

It is important to note that psychosocial risks also significantly affect patient safety. The World Health Organization (WHO) defines patient safety as the prevention of errors and adverse effects associated with healthcare. Millions of patients worldwide experience disabilities, injuries, or death due to unsafe medical practices each year [21]–[24]. There is a recognized relationship between patient safety, staff well-being, and organizational culture. Improving this relationship benefits both patients and healthcare workers [25],[26].

Studying organizational culture can be approached by examining the entire organization or by analyzing hierarchical levels or professional groups. These approaches provide valuable insights into how healthcare systems operate and how they can be improved to achieve better outcomes [27]–[30]. When focusing on the organization as a whole, it is important to consider shared values, perceptions, and behavior patterns that shape the work environment. Organizational culture influences employee performance, service quality, and overall workplace atmosphere. In healthcare, a positive organizational culture fosters open communication, collaboration, and continuous improvement, which enhances both patient safety and staff well-being [31]–[33].

Psychosocial risks are also closely tied to medication errors. Medication errors are among the most common types of errors in healthcare, leading to significant economic costs and negatively impacting patients' quality of life [34]. Many definitions of medication errors exist in the literature, with the first formal definition provided by the American Hospital Association in 1954. The National Coordinating Council for Medication Error Reporting and Prevention (NCCMERP) defines medication errors as any preventable event that may cause or lead to inappropriate medication use or harm while the medication is under the control of a healthcare professional or patient [35],[36]. Over the years, medication errors have been categorized into various types, including wrong patient, wrong dose, or wrong administration time [37]–[40].

Nurses are traditionally trained to follow the “five rights” of medication administration: right medication, right dose, right route, right time, and right patient. However, these guidelines are often insufficient, as they focus on the final stage of administration and do not account for interdisciplinary responsibility or the complexity of the task. Medication errors can result in prolonged hospital stays, additional interventions, and life-threatening situations [41]–[44].

Therefore, preventing these errors should be a top priority in healthcare [45],[46]. Our aim of this study is to know the psychosocial risks experienced by nurses in tertiary hospitals in Greece and their association with their attitudes towards safety and the occurrence of medication errors.

## 2. Materials and methods

### 2.1. Study design

A cross-sectional study, based on a self-reported questionnaire, was conducted at General University Hospital of Larissa, General Hospital of Thessaloniki G. Papanikolaou, General Hospital of Nice Agios Panteleimon and Athens General Hospital O Evangelismos.

The selection of the aforementioned hospitals was carried out by initially identifying all tertiary hospitals throughout Greece, from which those with more than 500 beds were selected. Additionally, the number of hospitalized patients exceeded 28,000 annually, and the days of hospitalization exceeded 100,000 per year. Based on the aforementioned criteria, we created three zones. Zone A included hospitals with more than 500 beds, more than 60,000 hospitalized patients per year, and more than 200,000 days of hospitalization per year. Zone B included hospitals with more than 500 beds, 28,000–35,000 hospitalized patients per year, and more than 100,000 days of hospitalization per year. Zone C included hospitals with more than 500 beds, 35,000–70,000 hospitalized patients per year, and 100,000–200,000 days of hospitalization per year.

Based on these created zones, within each health region the largest and smallest hospitals that met the inclusion criteria were selected to ensure a diverse and representative sample for the study. It should be noted that in Greece, there are seven Health Regions (Attica, Piraeus and the Aegean, Macedonia, Macedonia and Thrace, Thessaly and Central Greece, Peloponnese/Ionian Islands/Epirus/Western Greece, Crete), each encompassing all the healthcare services of the country.

The hospitals selected for the study belong to the following Zones and Health Regions: (a). Evangelismos Hospital belongs to the 1^st^ Health Region and meets the criteria of Zone A. (b). Nikaia Hospital belongs to the 2^nd^ Health Region and meets the criteria of Zone B. (c). Papanikolaou Hospital belongs to the 3^rd^ Health Region and meets the criteria of Zone C. (d). The University Hospital of Larissa belongs to the 5^th^ Health Region and meets the criteria of Zone C.

The final selection was made taking into account the geographical location of each hospital as well as ensuring the participation of the majority of Health Regions, with four of the seven Health Regions participating in the study. Additionally, the hospitals selected are also the largest in their respective Health Regions.

### 2.2. Eligibility criteria

The participants were nurses who met the inclusion and exclusion criteria. More specifically, the inclusion criteria included: Being a nurse, working in their current position for at least 12 months, being able to read and understand the Greek language, and being aged between 20 and 67 years. Exclusion criteria included nurses employed for less than 12 months, those in administrative roles with no direct patient contact, and those who were on leave during the study period.

### 2.3. Ethical issues

For the conduct of the research, permissions were obtained from the Scientific Council of each hospital. Participants were informed about the purpose of the research, the preservation of anonymity, the voluntary nature of their participation, and their right to refuse. Consent was obtained from all participants, and permissions from the creators of the research tools used in the study were received.

### 2.4. Data collection/instrument

Data collection was carried out through the completion of questionnaires by nursing staff who meet the participation criteria. The questionnaire was provided to participants in both printed and electronic form (Google Forms) at their workplace. Of the 600 printed questionnaires distributed, 500 were returned fully completed, while only 14 questionnaires were completed via the online form. Incomplete questionnaires were excluded from the analysis to maintain data integrity. To ensure that each electronic response was unique, the option for respondents to sign in using their Google accounts was activated, enabling only one response per account.

The questionnaire included demographics of nursing staff (age, gender, marital status, work experience, level of education), characteristics of the nursing unit (pathology, surgical clinic, years of service, Intensive Care Unit), the COPSOQ III questionnaire [47]–[50], the validated Greek version (Copenhagen Psychosocial Questionnaire Version III), the HSOPSC (Hospital Survey on Patient Safety Culture) questionnaire [51],[52], the validated Greek version, and the questionnaire for investigating nursing errors in drug administration [53]. Additionally, a website (https://vasileiostzen.wixsite.com/psycosocial-risks) has been created exclusively for the purposes of the study, where each participant has their own separate profile and can access details about the subject as well as the progress of the research. The collection of questionnaires was carried out during the years 2022 and 2023.

### 2.5. Statistical analysis

Quantitative variables were expressed as mean values (Standard Deviation) and as median (interquartile range), while categorical variables were expressed as absolute and relative frequencies. Mann-Whitney test was used for the comparison of HSOPSC and COPSOQ III subscales between participants who had realized that they had done an error in medication administration and those who had not done such an error.

A hierarchical logarithmic regression was performed with whether participants perceived they had made a mistake as the dependent variable. In the 1^st^ step of the analysis, the demographic data of the participants was entered, using the enter method. In the 2^nd^ step, job-related factors were entered, using the enter method. In the 3^rd^ step, the dimensions of the psychosocial risks scale were entered, using the stepwise method (*p* for entry 0.05, *p* for removal 0.10). In the 4^th^ step, the dimensions of the safety scale were entered, using the stepwise method (*p* for entry 0.05, *p* for removal 0.10). Odds ratios (*OR*) with their 95% confidence intervals (95% *CI*) were computed from the results of the logistic regression analyses.

For the investigation of the mediating role of safety in the association between psychosocial risk factors and realizing that an error in medication administration had occurred, SPSS PROCESS macro was used following Hayes guidelines. A 5000-sample boot-strap procedure was used to estimate bias-corrected 95% confidence intervals (*CI*s) to test the significance of indirect effect of the relationships. Mediation is presented when the indirect effect is significant, i.e., if confidence intervals do not contain zero. According to Hayes [54] and colleagues [55],[56] this bootstrapping procedure overcomes the limitations of the approaches highlighted by Baron et al. [57] and Sobel [58] yielding results that are more accurate and less affected by sample size. Full mediation is presented when the direct effect is not significant, while partial mediation is presented when the direct effect is significant.

All reported *p* values are two-tailed. Statistical significance was set at *p* < 0.05 and analyses were conducted using SPSS statistical software (version 26.0).

## 3. Results

The sample consisted of 514 nurses, representative of the total population, whose characteristics are presented in Table 1. The sample size was calculated using a confidence interval of 95% and a margin of error of 5%, ensuring that the final sample adequately represents the population of nurses in Greek tertiary hospitals. Additionally, the distribution of questionnaires was proportionate to the size of the hospitals based on the number of nursing staff in each institution. Females made up 74% of the participants, and 42.2% were aged 26–35 years. Additionally, 63.2% of the participants were married, and 17.4% held an MSc degree. Moreover, 52.3% of the participants worked in rotating shifts. An electronic medication management system was present in the department of 52.3% of the participants. According to 37.4% of the participants, in-hospital continuing education programs were often implemented, while 35.6% reported that such programs were rarely implemented. The workload for 53.9% of the sample was very heavy, and 42% experienced a high degree of burnout. Average staffing was reported by 40.3% of the participants, while 20.6% indicated poor staffing levels.

Table 2 presents the work-related data of the participants. Specifically, 33.1% worked at Papanikolaou General Hospital of Thessaloniki, while 25.9% were employed at Evangelismos Hospital. The average length of employment at the current hospital was 15 years (*SD* = 9.7 years), and in the current department, 8.4 years (*SD* = 6.6 years), while the average total work experience was 16.6 years (*SD* = 9.9 years). The median number of afternoon and night shifts was 2 (range: 2–2). Almost all participants (99.4%) had direct contact with patients. The average number of beds per department was 28.4 (*SD* = 14.5), while the average number of nurses was 20.6 (*SD* = 10.4). The median number of nurses on the morning shift was 4 (range: 4–7), on the afternoon shift 3 (range: 2–3), and on the night shift 2 (range: 2–3). A total of 86.4% of participants did not work overtime. Additionally, 98.4% of nurses were involved in medication administration during the morning shift, and 96.9% during the afternoon/night shifts. On average, participants spent 14.5% of their time on paperwork during the morning shift (*SD* = 9.7%). Finally, 18.9% of nurses worked in an environment with a policy against harassment and violence, while 24.7% worked in a space with a gender discrimination policy.

**Table 1.** Demographical and work-related characteristics of the sample.

**Project**		***n* (%)**
Gender	
	Men	127 (26.0)
	Women	362 (74.0)
Age (years)		
	18–25	84 (16.3)
	26–35	217 (42.2)
	36–45	154 (30.0)
	46–55	57 (11.1)
	56–65	2 (0.4)
Family status		
	Unmarried	122 (23.7)
	Married	325 (63.2)
	Divorced	67 (13.0)
Educational level		
	Secondary	160 (34.7)
	Technological university	212 (46.0)
	University	6 (1.3)
	MSc	80 (17.4)
	PhD	3 (0.7)
Total working experience (years), *mean* (*SD*)		16.6 (9.9)
Number of nurses in the department, median (IQR)		18 (14–20)
Working overtime during the week		70 (13.6)
What percentage of your time do you spend on paperwork during the morning shift? *mean* (*SD*)		14.5 (9.7)
Working in rotated shifts		269 (52.3)
Is there an electronic management system for medication administration in your department?		269 (52.3)
How often are continuing education programs implemented in the hospital?		
	Always	10 (1.9)
	Very often	129 (25.1)
	Often	192 (37.4)
	Rarely	183 (35.6)
	Never	0 (0)
The workload in your department is:		
	Very high	277 (53.9)
	High	193 (37.5)
	Moderate	44 (8.6)
	Low	0 (0)
	Very low	0 (0)
	Very high	143 (27.8)
The degree of burnout you feel is:		
	High	216 (42.0)
	Moderate	128 (24.9)
	Low	20 (3.9)
	Very low	7 (1.4)
	Excellent	7 (1.4)
Do you think staffing in your department is:		
	Very good	88 (17.1)
	Good	106 (20.6)
	Moderate	207 (40.3)
	Bad	106 (20.6)
How often are you distracted or interrupted by extraneous factors while administering medication?		
	Always	35 (6.8)
	Very often	241 (46.9)
	Often	174 (33.9)
	Rarely	64 (12.5)
	Never	0 (0)
Do you apply critical thinking before carrying out medical medication instructions, for their correctness?		
	Always	199 (38.7)
	Very often	146 (28.4)
	Often	163 (31.7)
	Rarely	6 (1.2)
	Never	0 (0)

**Table 2.** Occupational characteristics of participants.

**Project**		***n***	**%**
Occupational Hospital			
	Agios Panteleimon Regional General Hospital of Nikaia	115	22.4
	Papanikolaou hospital	170	33.1
	Evangelismos	133	25.9
	Larissa General Hospital	96	18.7
Length of time (years) in this hospital		*Mean* (*SD*)	*Median* (*R*)
		15 (9.7)	14 (7–22)
Time interval (years) in this segment		*Mean* (*SD*)	*Median* (*R*)
		8.4 (6.6)	7 (3–12)
Years of total service		*Mean* (*SD*)	*Median* (*R*)
		16.6 (9.9)	16 (9–24)
Number of afternoon shifts per week		*Mean* (*SD*)	*Median* (*R*)
		2 (0.5)	2 (2–2)
Number of night shifts week		*Mean* (*SD*)	*Median* (*R*)
		1.8 (0.5)	2 (2–2)
Have direct communication with the patient			
	No	3	0.6
	Yes	511	99.4
Number of beds		*Mean* (*SD*)	*Median* (*R*)
		28.4 (14.5)	25 (18–39)
Number of nurses in the Department		*Mean* (*SD*)	*Median* (*R*)
		20.6 (10.4)	18 (14–20)
Educational level		*Mean* (*SD*)	*Median* (*R*)
	Technological university	1.2 (1.7)	1 (0–2)
	University	11.7 (6.0)	10 (8–13)
	Secondary	7.7 (5.4)	6 (4–10)
People on the morning shift (excluding supervisor)		*Mean* (*SD*)	*Median* (*R*)
		5.6 (3.0)	4 (4–7)
Number of nurses in the afternoon shift		*Mean* (*SD*)	*Median* (*R*)
		3.1 (1.7)	3 (2–3)
Number of nurses in night shift		*Mean* (*SD*)	*Median* (*R*)
		2.5 (1.2)	2 (2–3)
Overtime per week			
	At all	444	86.4
	1–2 times a week	46	8.9
	3 times a week	6	1.2
	Daily	18	3.5
Hours overtime daily		*Mean* (*SD*)	*Median* (*R*)
		0.13 (0.38)	0 (0–0)
Are you involved in administering medication during the morning shift?			
	No	8	1.6
	Yes	506	98.4
Are you involved in administering medication during the afternoon/evening shift?			
	No	16	3.1
	Yes	498	96.9
How much of your time (hours) do you spend on paperwork during the morning shift		*Mean* (*SD*)	*Median* (*R*)
		14.5 (9.7)	10 (10–20)
Does your workplace have a policy against harassment and violence?			
	No	417	81.1
	Yes	97	18.9
Does your workplace have a policy against gender discrimination?			
	No	387	75.3
	Yes	127	24.7

Regarding medication administration errors in the past year, 64.4% of the sample realized they had made an error. Moreover, 55.4% of the participants reported that they had rarely recognized making such a mistake in the past 12 months (Table 3). Additionally, 33.7% of the participants believed that medication errors were related to administering the wrong drug, and 16% attributed errors to the wrong dose. Furthermore, 62.1% of the participants had never reported an actual medication error in the past 12 months. A total of 34.0% believed that most mistakes occurred during the afternoon shift, while 26.1% felt there was no difference between shifts. Moreover, 52.3% of participants reported the error to the physician, and 29.6% to the supervisor. A total of 54.7% of participants addressed their errors by trying to improve their training. Errors were typically dealt with through discussions, both with supervisors (77.6%) and colleagues (73.0%). Additionally, 41.9% of participants hid their mistakes due to fear of negative comments, and 59.0% because of feeling guilty.

**Table 3.** Information on errors about medication administration.

**Project**		***n* (%)**
In the past 12 months, how often have you realized that you have made mistakes in the administration of medicines?		
	Daily	0 (0.0)
	Very often	0 (0.0)
	Often	46 (8.9)
	Rarely	285 (55.4)
	Never	183 (35.6)
	Wrong patient	15 (2.9)
	Wrong medicine	173 (33.7)
		
You consider medication errors to mainly concern:		
	Incorrect administration time	79 (15.4)
	Wrong dose	82 (16.0)
	Missed dose	77 (15.0)
	Incorrect route of administration	30 (5.8)
	Administration of additional dose	26 (5.1)
	Incorrect drug dissolution	6 (1.2)
	Other	26 (5.1)
How often in the last 12 months have you been told an actual medication error?		
	Daily	0 (0.0)
	Very often	0 (0.0)
	Often	23 (4.5)
	Rarely	172 (33.5)
	Never	319 (62.1)
At what time of day have you noticed that most medication errors occur?		
	Morning shift	45 (8.8)
	Afternoon shift	175 (34)
	Night shift	85 (16.5)
	No difference in shifts	134 (26.1)
	I do not know	75 (14.6)
In case you make a mistake, report it mainly:		
	To the supervisor	152 (29.6)
	To the doctor	269 (52.3)
	To a colleague	64 (12.5)
	To a friend	0 (0.0)
	In the family	0 (0.0)
	To nobody	29 (5.6)
	A problem with working conditions	220 (42.8)
If you make a mistake, take it personally with:		
	Trying to improve your training	281 (54.7)
	Indifference	7 (1.4)
	Other	6 (1.2)
Your supervisor usually deals with your mistakes by:		
	Discussion	399 (77.6)
	Indifference	73 (14.2)
	Reproach	42 (8.2)
	Other	0 (0.0)
Your colleagues usually deal with your mistakes by:		
	Discussion	375 (73.0)
	Indifference	78 (15.2)
	Negative comments	55 (10.7)
	Other	6 (1.2)
	Try to justify	13 (2.5)
In case you realize a mistake of yours, your first feeling is:		
	Anger	168 (32.9)
	Guilt	301 (59.0)
	Indifference	0 (0)
	Other	28 (5.5)
	The negative comments	212 (41.9)
The most important reason to hide a mistake of yours, you think is:		
	The sanctions	48 (9.5)
	Your judgment that it is not important to mention	215 (42.5)
	Other	31 (6.1)

Table 4 presents the scores on COPSOQ III dimensions for the total sample and according to whether or not they realized they had made a medication error in the past 12 months. Scores on the dimensions ‘Quantitative Demands’, ‘Bullying’, ‘Gossip and Slander’, ‘Conflicts and Quarrels’, ‘Sexual Harassment’, ‘Physical Violence’, ‘Sense of Community at Work’, and ‘Physical Work Environment’ were significantly higher in participants who realized they had made a medication error in the past 12 months. Conversely, scores on the dimensions ‘Variation of Work’, ‘Meaning of Work’, ‘Predictability’, ‘Social Support from Colleagues’, ‘Bullying from Customers (External Persons)’, and ‘Work Engagement’ were significantly lower in participants who had realized they had made a medication error in the past 12 months.

**Table 4.** Scores in COPSOQ III scales, in total sample and by having realized that an error in the medication administration had occurred.

**Project**	**Total Median (IQR)**	**No Error Median (IQR)**	**Error Median (IQR)**	***p* Mann-Whitney test**
Work pace	75 (62.5–75.0)	75 (62.5–75.0)	75 (62.5–75.0)	0.787
Quantitative demands	33.3 (16.7–50.0)	25 (16.7–33.3)	33.3 (25.0–50.0)	<0.001
Emotional demands	62.5 (50.0–75.0)	62.5 (50.0–75.0)	62.5 (50.0–75.0)	0.249
Demands for hiding emotions	50 (37.5–62.5)	50 (50.0–62.5)	62.5 (37.5–62.5)	0.341
Work life conflict	35.7 (25.0–50.0)	32.1 (25.0–50.0)	35.7 (25.0–53.6)	0.154
Influence	58.3 (41.7–66.7)	58.3 (41.7–66.7)	58.3 (41.7–66.7)	0.665
Control over working time	50 (37.5–62.5)	50 (25.0–62.5)	37.5 (37.5–62.5)	0.588
Variation of work	50 (25.0–50.0)	50 (25.0–75.0)	50 (25.0–50.0)	0.020
Possibilities for development	25 (25.0–50.0)	25 (25.0–50.0)	25 (25.0–37.5)	0.114
Meaning of work	25 (0.0–50.0)	25 (12.5–62.5)	25 (0–37.5)	<0.001
Commitment to the workplace	25 (12.5–62.5)	25 (25.0–62.5)	25 (12.5–62.5)	0.087
Predictability	37.5 (25.0–62.5)	37.5 (25.0–62.5)	37.5 (25.0–50.0)	0.048
Role clarity	25 (16.7–41.7)	25 (16.7–41.7)	25 (16.7–41.7)	0.116
Role conflicts	58.3 (41.7–66.7)	58.3 (41.7–66.7)	58.3 (50.0–75.0)	0.059
Quality of leadership	37.5 (25.0–50.0)	37.5 (25.0–50.0)	43.8 (25.0–50.0)	0.658
Social support from colleagues	66.7 (58.3–66.7)	66.7 (66.7–75)	66.7 (58.3–66.7)	<0.001
Bullying	50 (37.5–62.5)	50 (37.5–50.0)	50 (50.0–62.5)	<0.001
Gossip and slander	75 (50.0–75.0)	75 (50.0–75.0)	75 (50.0–100)	0.006
Conflicts and quarrels	75 (50.0–75.0)	75 (50.0–75.0)	75 (50.0–100)	0.012
Unpleasant teasing	50 (25.0–75.0)	50 (25.0–75.0)	50 (25.0–75.0)	0.244
Cyber bullying	50 (25.0–75.0)	50 (25.0–75.0)	50 (50.0–75.0)	0.078
Sexual harassment	50 (50.0–75.0)	50 (50.0–75.0)	50 (50.0–75.0)	0.028
Threats of violence	75 (50.0–100)	75 (50.0–100.0)	75 (50.0–100)	0.115
Physical violence	75 (50.0–100)	75 (50.0–100.0)	75 (75.0–100)	0.042
Social support from supervisor	93.8 (87.5–100)	93.8 (81.3–100.0)	87.5 (87.5–100)	0.177
Sense of community at work	100 (75.0–100)	100 (75.0–100.0)	100 (100–100)	0.044
Vertical trust	50 (25.0–62.5)	50 (25.0–75.0)	50 (25.0–62.5)	0.384
Organizational justice	50 (37.5–62.5)	50 (37.5–50.0)	50 (25.0–62.5)	0.062
Recognition	50 (25.0–75.0)	50 (50.0–75.0)	50 (25.0–75.0)	0.400
Physical Work Environment	37.5 (29.2–50.0)	33.3 (25.0–54.2)	41.7 (29.2–50.0)	0.009
Job Insecurity	16.7 (0–25.0)	8.3 (0–25.0)	16.7 (0–25.0)	0.235
Insecurity over working conditions	33.3 (16.7–50.0)	33.3 (0–50.0)	33.3 (16.7–50.0)	0.617
Bullying from customers	32.5 (17.5–42.5)	37.5 (17.5–42.5)	30 (15.0–40.0)	0.024
(External Persons)				
Personal wellbeing	50 (33.3–58.3)	50 (33.3–58.3)	50 (33.3–58.3)	0.358
Intention to leave	37.5 (12.5–50.0)	37.5 (25–50)	37.5 (12.5–50.0)	0.371
Job satisfaction	53.6 (35.7–60.7)	50 (32.1–57.1)	53.6 (35.7–60.7)	0.156
Self rated health	20 (10.0–40.0)	20 (20–30)	30 (10.0–40.0)	0.708
Burnout	50 (45.0–65.0)	55 (45–62.5)	50 (45.0–70.0)	0.182
Work engagement	41.7 (25.0–50.0)	41.7 (33.3–50.0)	33.3 (25.0–50.0)	**0.026**
Mobbing	5 (0–15.0)	5 (0–15.0)	10 (0–15.0)	0.267

Table 5 presents the scores on the dimensions of the HSOPSC scale for the total sample and according to whether or not they realized they had made a medication error in the past 12 months. Scores on the dimensions ‘Teamwork Within Units’, ‘Organizational Learning—Continuous Improvement’, ‘Feedback & Communication About Error’, ‘Communication Openness’, ‘Teamwork Across Units’, and ‘Handoffs & Transitions’ were significantly higher in participants who realized they had made a medication error in the past 12 months. In contrast, the score on the ‘Staffing’ dimension was significantly lower in participants who realized they had made a medication error in the past 12 months.

**Table 5.** Scores in HSOPSC scales. in total sample and by having realized that an error in the medication administration had occurred.

**Project**	**Total Median (IQR)**	**No Error Median (IQR)**	**Error Median (IQR)**	***p* Mann-Whitney test**
Teamwork Within Units	50 (25.0–75.0)	50 (0–75.0)	75 (25.0–75.0)	<0.001
Supervisor/Manager Expectations & Actions Promoting Patient Safety	50 (25.0–75.0)	50 (25.0–100)	50 (25.0–75.0)	0.398
Organizational Learning—Continuous Improvement	33.3 (0–66.7)	33.3 (0–66.7)	33.3 (0–66.7)	<0.001
Management Support for Patient Safety	0 (0–33.3)	0 (0–66.7)	0 (0–33.3)	0.792
Overall Perceptions of Patient Safety	50 (0–75.0)	50 (0–75.0)	50 (25.0–75.0)	0.141
Feedback & Communication About Error	33.3 (0–66.7)	33.3 (0–66.7)	66.7 (0–100)	<0.001
Communication Openness	33.3 (33.3–66.7)	33.3 (33.3–66.7)	33.3 (33.3–66.7)	0.015
Frequency of Events Reported	0 (0–66.7)	0 (0–33.3)	0 (0–100)	0.098
Teamwork Across Units	37.5 (25.0–75.0)	25 (25.0–50.0)	50 (25.0–75.0)	0.022
Staffing	25 (0–50.0)	25 (25.0–50.0)	25 (0–25.0)	<0.001
Handoffs & Transitions	50 (25.0–75.0)	50 (25.0–75.0)	50 (25.0–75.0)	0.001
Nonpunitive Response to Errors	33.3 (0–33.3)	33.3 (0–33.3)	33.3 (0–33.3)	0.895

Demographics were not significantly related to whether participants perceived they had made a mistake (Table 6). From the work-related information, it was found that the more nurses there were, the more likely they were to realize they had made a mistake in medication administration (*OR* = 1.03, *p* = 0.041). Additionally, the more frequently continuing education programs were implemented in the hospital, the lower the probability of making a mistake (*OR* = 0.58, *p* = 0.001), and the less often they engaged in critical thinking before executing medical medication instructions, the lower the probability of error (*OR* = 0.53, *p* < 0.001). In the third step, the dimensions ‘Quantitative Demands’ (*OR* = 1.02, *p* = 0.015) and ‘Bullying’ (*OR* = 1.02, *p* = 0.005) were identified as significant psychosocial risk factors, indicating that higher risks in these areas increased the likelihood of medication errors. However, when ‘Staffing’ and ‘Handoffs & Transitions’ were introduced as significant safety factors in the fourth step, the psychosocial risk factor ‘Quantitative Demands’ lost its significance, while ‘Bullying’ remained significant. The final conclusion regarding psychosocial risks and safety factors is that a higher risk of Bullying is associated with a greater probability of a medication error (*OR* = 1.02, *p* = 0.011), more safety in Staffing is associated with a lower likelihood of a medication error (*OR* = 0.98, *p* < 0.001), and more safety in Handoffs & Transitions is associated with a greater likelihood of a medication error (*OR* = 1.01, *p* = 0.001).

**Table 6.** Hierarchical logistic regression with having realized that an error in the medication administration had occurred as dependent variable.

**Project**		***OR* (95% *CI)*+**	***p***
	Gender (Women *vs*. Men)	0.75 (0.43–1.31)	0.307
Step1: Demographical data			
	Age	1.62 (0.95–2.77)	0.074
	Education Level^a^	0.82 (0.64–1.04)	0.104
	Family status		
	Unmarried *vs*. Married	1.27 (0.69–2.33)	0.439
	Divorced *vs*. Married	0.91 (0.44–1.88)	0.800
	Total working experience (years)	0.96 (0.91–1.00)	0.065
	Number of nurses in the department	1.03 (1.01–1.05)	**0.041**
Step 2: Work-related information			
	Working overtime during the week	0.98 (0.49–1.96)	0.947
	Percentage of Time on Paperwork During Morning	1.00 (0.97–1.03)	0.933
	Shift		
	Working in rotated shifts (Yes *vs*. No)	0.64 (0.33–1.24)	0.187
	Electronic Medication Management System in Department (Yes *vs*. No)	0.85 (0.47–1.54)	0.585
	Continuing Education Programs Frequency^1^	0.58 (0.42–0.81)	**0.001**
	Workload in Department:^2^	0.77 (0.51–1.18)	0.231
	Degree of Burnout:^2^	0.86 (0.62–1.19)	0.373
	Staffing Quality in Department^3^	1.19 (0.89–1.60)	0.241
	Frequency of Distractions	0.96 (0.69–1.34)	0.830
	During Medication Administration^1^		
	Critical Thinking Before Medication Administration^1^	0.53 (0.40–0.71)	**<0.001**
Step 3: COPSOQ subscales			
	Quantitative Demands	1.02 (1.00–1.03)	0.066
	Bullying	1.02 (1.01–1.03)	**0.011**
Step 4: HSOPSC subscales			
	Staffing	0.98 (0.96–0.99)	**<0.001**
	Handoffs & Transitions	1.01 (1.01–1.02)	**0.001**

Note: +: Relative ratio (95% Confidence Interval); ^a^: ranging from 1 (secondary education) to 5 (doctoral degree); ^1^: Range of values from 1 (always) to 5 (never); ^2^: Range of values from 1 (very large) to 5 (very small); ^3^: Range of values from 1 (excellent) to 5 (poor).

Through the PROCESS procedure, which investigates the mediating role of safety in the relationship between psychosocial risks and medication errors, it was found that the safety factors ‘Staffing’ and ‘Handoffs & Transitions’ partially mediate the relationship between ‘Quantitative Demands’ and medication error occurrence (significant indirect effect ‘Staffing’ = 0.0074 with 95% CI: 0.0030–0.0125, significant indirect effect ‘Handoffs & Transitions’ = −0.0037 with 95% *CI*: −0.0063–−0.0018, and significant direct effect = 0.0279 with 95% *CI*: 0.0162–0.0397). However, there is no mediation in the relationship between ‘Bullying’ and medication errors (non-significant indirect effect of ‘Staffing’ = 0.0004 with 95% *CI*: −0.0024–0.0037, non-significant indirect effect of ‘Handoffs & Transitions’ = 0.0000 with 95% *CI*: −0.0017–0.0016, and significant direct effect = 0.0265 with 95% *CI*: 0.0139–0.0392).

## 4. Discussion

In this study, we aimed to investigate the psychosocial risks experienced by nurses in tertiary hospitals in Greece and their association with attitudes towards safety and the occurrence of medication errors. The results of our study underscore several critical findings that significantly contribute to the existing body of literature on occupational health in healthcare settings. Primarily, the analysis indicated that nurses exposed to elevated levels of psychosocial risks, including ‘Quantitative Demands’, ‘Bullying’, ‘Gossip and Slander’, ‘Conflicts and Quarrels’, ‘Sexual Harassment’, ‘Physical Violence’, ‘Sense of Community at Work’, and ‘Physical Work Environment’, exhibited a higher propensity to report medication errors. These findings align with Brennan (2017), who emphasized that high levels of workplace stress and exposure to psychosocial risks undermine nurses' resilience and well-being, making them more vulnerable to errors [8]. Similarly, Garcia et al. (2019) highlighted that burnout, often a consequence of sustained stress, has a direct negative impact on patient safety by impairing cognitive functions and decision-making processes [9]. Together, these studies corroborate the notion that high-stress environments and adverse workplace interactions markedly increase the likelihood of errors. Conversely, dimensions such as ‘Variation of Work’, ‘Meaning of Work’, ‘Predictability’, ‘Social Support from Colleagues’, ‘Bullying from Customers (External Persons)’, and ‘Work Engagement’ were markedly lower among participants who reported medication errors. This suggests that a supportive and predictable work environment, where nurses derive meaning from their work and receive sufficient social support, is paramount in minimizing errors. These findings corroborate the theoretical framework positing that job satisfaction and a positive work environment are fundamental to the provision of high-quality healthcare [15],[16].

It was also determined that organizational factors play a significant role in influencing medication errors. Participants who perceived higher levels of ‘Teamwork Within Units’, ‘Organizational Learning—Continuous Improvement’, ‘Feedback and Communication About Error’, ‘Communication Openness’, ‘Teamwork Across Units’, and ‘Handoffs and Transitions’ were less likely to report errors. These findings under-score the importance of fostering a collaborative and open communication culture within healthcare settings to enhance patient safety [59],[60]. Interestingly, the dimension of ‘Staffing’ was found to be significantly lower among those who reported errors, suggesting that inadequate staffing levels are a critical risk factor for medication errors. This finding aligns with Upadhyay et al. (2022), who highlighted that cultural competency and staffing levels are pivotal in shaping patient safety outcomes. Their study demonstrated that better staffing ratios are associated with reduced error rates and improved patient outcomes, emphasizing the critical role of staffing adequacy in healthcare systems [27].

Through hierarchical logistic regression analysis, ‘Quantitative Demands’ and ‘Bullying’ emerged as significant psychosocial risk factors. However, when ‘Staffing’ and ‘Handoffs and Transitions’ were incorporated as safety factors, ‘Quantitative Demands’ lost its significance, whereas ‘Bullying’ remained significant. This indicates that, although workload is an important factor, the impact of bullying is more pervasive and enduring. Consequently, targeted interventions are required to address workplace bullying and promote a safe and supportive environment [11].

The mediation analysis further elucidated the complex interplay between psychosocial risks and safety factors. ‘Staffing’ and ‘Handoffs & Transitions’ were found to partially mediate the relationship between ‘Quantitative Demands’ and medication error occurrence, underscoring the multifaceted nature of these issues. Effective management of staffing and transitions can mitigate the adverse effects of high work demands on error rates.

Overall, our findings underscore the critical need for comprehensive strategies that simultaneously address psychosocial risks and organizational safety factors to enhance both patient safety and nurse well-being. Implementing policies that ensure adequate staffing levels is paramount, as insufficient staffing has been directly linked to increased error rates and diminished quality of care. Promoting teamwork within and across units is equally essential; fostering a collaborative environment where nurses can rely on their colleagues for support can significantly reduce stress and prevent errors. Nurses per patient and workload are well-documented factors that influence medication errors and nurse well-being. Research suggests that higher ratios and increased workloads contribute to fatigue, burnout, and higher error rates [27]. In the context of Greece, where nurse-to-patient ratios are often lower than in other European countries, this may further exacerbate the risk of errors. Future research should control for these variables to better understand their impact on patient safety and nurse performance.

Continuous education and professional development programs are vital to keep nursing staff updated on the latest best practices and advancements in healthcare, thereby enhancing their competence and confidence in medication administration. Additionally, fostering a positive organizational culture that prioritizes open communication, mutual respect, and employee well-being is crucial. Such a culture not only enhances job satisfaction and reduces turnover rates but also improves overall patient care quality.

The Greek healthcare system, influenced by the economic crisis, faces challenges such as lower staffing levels and resource constraints, which may significantly affect the generalizability of study findings compared to other European countries. While reforms have aimed to strengthen public health services, Greece continues to spend less per capita on healthcare than the EU average, impacting the quality and accessibility of care [61]. In contrast, many European countries have higher healthcare spending and better nurse-to-patient ratios, contributing to more robust healthcare outcomes. Therefore, caution should be taken when generalizing our results to healthcare systems with different structures and resources.

Furthermore, there are limitations of its cross-sectional design, which restricts the ability to establish causal relationships between psychosocial risks and medication errors. Cross-sectional data capture associations at a single point in time, but they do not reveal how these relationships evolve. To better understand the causal mechanisms, researchers should employ longitudinal designs, tracking changes over time to assess how psychosocial stressors influence medication errors in dynamic environments. Additionally, intervention-based studies would be valuable to test targeted strategies for reducing stress and improving safety outcomes in healthcare settings [62]–[64]. Further research is warranted to explore the longitudinal effects of these interventions. Long-term studies could provide valuable insights into how sustained improvements in these areas impact nurse outcomes, such as job satisfaction, mental health, and professional performance, as well as patient care quality, including error rates, patient satisfaction, and overall health outcomes. This comprehensive approach, combining immediate interventions with ongoing research, can help create safer, more effective healthcare environments for patients and healthcare providers.

## 5. Conclusions

This study underscores the critical influence of psychosocial risks and organizational safety factors on the occurrence of medication errors among nurses in tertiary hospitals in Greece. The findings demonstrate that high levels of workplace stressors, such as quantitative demands and bullying, significantly correlate with an increased incidence of medication errors. Conversely, supportive and well-structured work environments characterized by effective teamwork, continuous learning, and open communication are associated with a reduction in these errors.

The necessity for comprehensive strategies that address both psychosocial and organizational aspects of the healthcare environment is evident. Moreover, ensuring adequate staffing levels is paramount, as it directly affects the workload and stress experienced by nurses. High workloads and inadequate staffing can lead to burnout and decreased vigilance, thereby increasing the likelihood of errors. By maintaining appropriate staffing levels, hospitals can alleviate some of the stressors that contribute to medication errors.

Moreover, fostering a positive organizational culture that promotes teamwork and open communication is essential. When nurses feel supported by their colleagues and have the opportunity to engage in continuous professional development, they are better equipped to handle the demands of their job effectively. This supportive environment not only improves job satisfaction and reduces turnover rates but also enhances the overall quality of patient care. Implementing policies that encourage open dialogue about errors without fear of retribution can lead to a more transparent and learning-focused culture, ultimately improving patient safety.

Interventions to mitigate workplace bullying are also crucial. Bullying creates a hostile work environment that can significantly impact mental health and job performance. Addressing bullying through targeted interventions can help create a safer and more supportive environment, reducing the occurrence of medication errors.

Researchers should explore the long-term effects of these interventions on both nurse outcomes and patient care quality. Longitudinal studies could provide valuable insights into how sustained improvements in workplace conditions impact job satisfaction, mental health, and professional performance of nurses, as well as patient safety and care quality. Understanding these long-term impacts can inform the development of more effective policies and practices that promote both nurse well-being and high-quality patient care.

In conclusion, this study highlights the multifaceted nature of the challenges faced by nurses in tertiary hospitals. Addressing both psychosocial risks and organizational safety factors through comprehensive strategies is essential for enhancing patient safety and nurse well-being. By focusing on adequate staffing, promoting a positive organizational culture, and mitigating workplace bullying, healthcare institutions can create safer and more effective work environments that benefit both healthcare providers and patients.

## Use of AI tools declaration

The authors declare they have not used Artificial Intelligence (AI) tools in the creation of this article.
